# Comparison of outcomes of neoadjuvant chemotherapy in *BRCA1*- versus *BRCA2*-associated breast and ovarian cancers

**DOI:** 10.37349/etat.2025.1002325

**Published:** 2025-06-18

**Authors:** Anna Sokolenko, Tatiana Gorodnova, Diana Enaldieva, Anna Shestakova, Alexandr Ivantsov, Anna Nyuganen, Igor Berlev, Petr Krivorotko, Alexey Belyaev, Evgeny Imyanitov

**Affiliations:** Scientific Director, IRCCS Istituto Romagnolo per lo Studio dei Tumori (IRST) “Dino Amadori”, Italy; ^1^Department of Tumor Growth Biology, N.N. Petrov Institute of Oncology, 197758 Saint-Petersburg, Russia; ^2^Department of Medical Genetics, St.-Petersburg State Pediatric Medical University, 194100 Saint-Petersburg, Russia

**Keywords:** *BRCA1*, *BRCA2*, breast cancer, pathologic complete response, neoadjuvant chemotherapy, ovarian cancer

## Abstract

**Aim:**

*BRCA1*/*2*-associated breast and ovarian carcinomas are often regarded as a single entity, assuming that *BRCA1* and *BRCA2* genes are almost equivalent with regard to their clinical significance. However, *BRCA1* and *BRCA2* genes differ in their function; therefore, a comparison of treatment outcomes in *BRCA1* vs. *BRCA2* carriers is warranted.

**Methods:**

This study focused on consecutive patients treated with neoadjuvant chemotherapy (NACT), given that these subjects are treatment-naive and accessible for immediate assessment of pathological and clinical outcomes.

**Results:**

*BRCA2*-associated high-grade serous ovarian carcinomas (HGSOCs) demonstrated significantly higher rates of pathologic complete response (pCR) as compared to *BRCA1*-related cancers [8/15 (53%) vs. 7/48 (15%), *P* = 0.004]. In contrast, HER2-negative breast cancer (BC) patients showed a numerically higher rate of pCR in *BRCA1* vs. *BRCA2* mutation carriers [38/69 (55%) vs. 13/36 (36%), *P* = 0.1]. However, the comparison with *BRCA-*wild-type (WT) tumors revealed that this tendency was mainly attributed to the increased prevalence of hormone receptor (HR)-negative disease in the former group. When BC patients were stratified according to the tumor receptor status, the response rates in triple-negative patients were consistently higher than in HR+/HER2– patients across all analyzed subgroups [*BRCA1*: 35/59 (59%) vs. 3/10 (30%); *BRCA2*: 5/10 (50%) vs. 8/26 (31%); WT: 31/76 (41%) vs. 12/74 (16%); Mantel-Haenzsel *P* < 0.001]. Logistic regression analysis revealed that the odds ratio (OR) for achieving pCR was higher for receptor status (triple-negative vs. HR+: OR = 3.4, 95% CI 1.9–6.0, *P* < 0.001) than for *BRCA* status (any mutation vs. WT: OR = 2.1, 95% CI 1.2–3.6, *P* = 0.008). The addition of carboplatin did not improve pCR rates in *BRCA1*- or *BRCA2*-associated BCs, while there was a numerically higher efficacy of carboplatin-containing regimens in patients with WT triple-negative tumors [14/26 (54%) vs. 15/44 (34%), *P* = 0.13].

**Conclusions:**

Hereditary ovarian carcinomas demonstrate better NACT outcomes in *BRCA2* vs. *BRCA1* mutation carriers. The opposite trend is observed in BC, which is likely to be attributed to a high frequency of triple-negative disease in *BRCA1*- but not *BRCA2*-associated BCs. Triple-negative receptor status rather than *BRCA1*/*2* status is the strongest predictor of response to NACT in BC.

## Introduction

*BRCA1* and *BRCA2* genes were discovered three decades ago via analysis of breast-ovarian cancer pedigrees. Subsequent studies revealed that they render essentially similar elevation in breast cancer (BC) and ovarian cancer (OC) risk. Hence, both these genes are considered almost equivalent when discussing the surveillance for healthy carriers or the feasibility of prophylactic surgery. Inactivation of both *BRCA1* and *BRCA2* is associated with homologous recombination deficiency (HRD) and, consequently, tumor sensitivity to PARP inhibitors, platinum compounds, and other drugs capable of inducing DNA double-strand breaks. In practical terms, *BRCA1*/*2* genes are commonly discussed as a single entity, and the differences between these two genes remain underappreciated.

Still, there are essential dissimilarities between *BRCA1* and *BRCA2* with regard to their biological functions, the spectrum of associated cancer types, and the nuances of tumor presentation. While both proteins are involved in DNA repair by homologous recombination, BRCA1 has a number of additional properties like ubiquitin-ligase activity and phosphorylation-dependent interaction with other proteins. It participates in the regulation of other than homologous recombination DNA repair pathways, cell cycle, transcription, R loop resolution, etc. [1]. Interestingly, BRCA1 appears to be essential for taxane-mediated apoptosis, whereas BRCA2 is not [2, 3]. Mutations in both *BRCA1* and *BRCA2* predispose to BC and OC, while a role in the development of prostate and pancreatic malignancies has been convincingly shown mainly for *BRCA2* but not for *BRCA1* [4–6]. With regard to BC, *BRCA1* is generally associated with receptor triple-negative disease, while *BRCA2*-related tumors usually express steroid hormone receptors (HRs) [7]. Furthermore, several clinical studies revealed differences between *BRCA1*- and *BRCA2*-mutated hereditary tumors with regard to the efficacy of various drug regimens and overall disease outcomes [8–11].

Neoadjuvant chemotherapy (NACT) is a commonly used approach aimed at reducing tumor volume before surgery. NACT is highly informative for clinical studies, as it involves relatively uniform groups of treatment-naive patients [12]. Furthermore, the post-NACT tumor pathologic response is regarded as the most informative surrogate of the true efficacy of a given drug combination [13]. This study aimed to compare NACT outcomes in *BRCA1*- vs. *BRCA2*-associated BC and OC patients.

## Materials and methods

The collection of hereditary high-grade serous ovarian carcinomas (HGSOCs) included 63 consecutive patients who carried *BRCA1* or *BRCA2* mutations and were subjected to NACT at the N.N. Petrov Institute of Oncology in the years 2019–2024. The majority of these patients were participants of a prospective clinical trial comparing the efficacy of mitomycin C plus cisplatin doublet vs. standard paclitaxel plus carboplatin regimen in *BRCA1*/*2* mutation carriers (NCT04747717, https://clinicaltrials.gov/). There were 48 patients with germline *BRCA1* mutations and 15 carriers of *BRCA2* pathogenic alleles (Table 1 and Table S1). Of these, six patients (three cases with *BRCA1* mutation and three cases with *BRCA2* mutation) did not undergo debulking surgery. The remaining 57 patients underwent interval cytoreduction with omentectomy. The WT (wild-type) arm included 93 consecutive germline *BRCA1*/*2* mutation-negative HGSOC patients eligible for NACT, who were treated with a standard paclitaxel plus carboplatin regimen within the years 2019–2024.

**Table 1:** Characteristics of OC patients (*n* = 156)

**Characteristics**	***BRCA1* (*n* = 48)**	***BRCA2* (*n* = 15)**	**WT (*n =* 93)**
Mean age (age range)	52.9 (36–70)	60.5 (43–76)	59.5 (28–83)
FIGO stage			
IIIC	21 (44%)	9 (60%)	46 (49%)
IVA	1 (2%)	1 (7%)	3 (3%)
IVB	26 (54%)	5 (33%)	44 (47%)
NACT regimen			
MP (mitomycin 10 mg/m^2^ + cisplatin 100 mg/m^2^, every 28 days)	26 (54%)	2 (13%)	-
TCbP (paclitaxel 175 mg/m^2^ + carboplatin AUC 6)	22 (46%)	13 (87%)	93 (100%)
RECIST 1.1			
Complete response	3 (6%)	1 (7%)	0 (0)
Partial response	33 (69%)	12 (80%)	62 (67%)
Stable disease	11 (23%)	1 (7%)	24 (26%)
Progressive disease	1 (2%)	1 (7%)	7 (8%)
CRS (omentum)			
CRS1	10 (21%)	1 (7%)	28 (30%)
CRS2	23 (48%)	2 (13%)	47 (51%)
CRS3, near-complete response	5 (10%)	1 (7%)	9 (10%)
CRS3, complete response	7 (15%)	8 (53%)	0 (0)
No debulking surgery/no omentectomy^※^	3 (6%)	3 (20%)	9 (10%)

^※^In the *BRCA1*/*2*-mutated subjects, four women (three *BRCA2* carriers and one *BRCA1* carrier) had an unresectable tumor after 3–4 cycles of NACT, one patient had a partial clinical response but was considered at high risk of post-operative complications due to comorbidities and continued on immunotherapy and olaparib, and one patient died during NACT due to pneumonia. In the WT group, all cases without debulking surgery were related to insufficient clinical response. AUC: area under the curve; CRS: chemotherapy response score; FIGO: International Federation of Gynecology and Obstetrics; MP: mitomycin and cisplatin; NACT: neoadjuvant chemotherapy; OC: ovarian cancer; RECIST: Response Evaluation Criteria In Solid Tumors; TCbP: carboplatin and paclitaxel; WT: wild-type

Pathologic responses were evaluated with the standard three-tier chemotherapy response score (CRS), which is routinely utilized for the assessment of NACT outcomes in women with HGSOC [14]. It is commonly accepted that CRS1 corresponds to no or minimal tumor response, CRS2 refers to multifocal or diffuse regression-associated changes with readily identifiable residual tumors, and CRS3 corresponds to a complete (no tumor cells) or near-complete response with minimal tumor foci up to a maximum of 2 mm [14].

BC study included consecutive patients who were treated with NACT within the years 2022–2024. Steroid HR status is a strong confounding factor for the response to NACT in BC, therefore, all comparisons considered both patients with *BRCA1*/*2* mutations and women with sporadic carcinomas. ER, PgR, and HER2 receptor status were determined as a part of regular BC histopathological diagnosis using treatment-naive tumor tissue. HER2-positive cases were excluded from the analysis as they all received anti-HER2 therapy. In total, the study included 105 *BRCA1* or *BRCA2* mutation carriers and 150 consecutive cases without germline pathogenic *BRCA1*/*2* variants. The majority of patients received standard anthracycline-taxane combinations. Carboplatin was added to the treatment scheme for some patients with *BRCA1*/*2*-associated and/or triple-negative disease, based on the physicians’ choice. 17 out of 255 patients received doxorubicin plus cyclophosphamide instead of taxane-containing regimens due to the preference of the doctor or the patient (Table 2).

**Table 2:** Characteristics of BC patients (*n* = 255)

**Characteristics**	***BRCA1* (*n* = 69)**	***BRCA2* (*n* = 36)**	**WT (*n* = 150)**
Mean age (age range)	42 (26–74)	43 (29–58)	46 (30–69)
UICC stage			
IA	4 (6%)	2 (6%)	9 (6%)
IIA	21 (30%)	5 (14%)	41 (27%)
IIB	23 (33%)	12 (33%)	47 (31%)
IIIA	9 (13%)	5 (14%)	27 (18%)
IIIB	3 (4%)	2 (6%)	4 (3%)
IIIC	9 (13%)	10 (28%)	22 (15%)
Receptor status			
Triple-negative	59 (86%)	10 (28%)	76 (51%)
Hormone receptor-positive	10 (14%)	26 (72%)	74 (49%)
Chemotherapy regimen			
AC + T	35 (51%)	24 (67%)	111 (74%)
AC + TCbP	31 (45%)	11 (31%)	26 (17%)
AC	3 (4%)	1 (3%)	13 (9%)
RECIST 1.1			
Complete response	28 (41%)	9 (25%)	25 (17%)
Partial response	37 (54%)	23 (64%)	98 (65%)
Stable disease	4 (6%)	4 (11%)	19 (13%)
Progressive disease	0 (0)	0 (0)	8 (5%)

AC + T, doxorubicin 60 mg/m^2^ and cyclophosphamide 600 mg/m^2^ (4 cycles) followed by 12 cycles of weekly paclitaxel 80 mg/m^2^; AC + TCbP, doxorubicin 60 mg/m^2^ and cyclophosphamide 600 mg/m^2^ (4 cycles) followed by 12 cycles of weekly paclitaxel 80 mg/m^2^ and carboplatin AUC 2; AC, doxorubicin 60 mg/m^2^ and cyclophosphamide 600 mg/m^2^ (4 cycles). AUC: area under the curve; BC: breast cancer; RECIST: Response Evaluation Criteria In Solid Tumors; UICC: Union for International Cancer Control; WT: wild-type

*BRCA1*/*2* full-length analysis covered coding regions of the genes and splice sites. DNA testing was performed using targeted NGS (next-generation sequencing) [15, 16].

The analysis of NACT efficacy in BC patients usually relies on the residual cancer burden (RCB) score, where RCB 0 indicates pathologic complete response (pCR), RCB I corresponds to minimal residual disease, RCB II reflects moderate residual disease, and RCB III refers to extensive residual tumor [17]. Detailed patient characteristics are presented in Table 2 and Table S2.

Statistical analysis was performed using SPSS Statistics (version 26.0). Tumor response frequencies were compared by Fisher’s exact test and Mantel-Haenzsel method. All statistical comparisons were two-tailed, with a 5% threshold (*P* = 0.05) for statistical significance. Odds ratios (ORs) were calculated using the logistic regression function.

## Results

### High rate of pCRs in *BRCA2*-associated HGSOCs

*BRCA1*-associated HGSOCs accounted for 48/63 (76%) patients with hereditary disease, while only 15/63 (24%) women were *BRCA2* mutation carriers. This is an expected distribution, given that the majority of *BRCA* mutations in Slavic countries are represented by *BRCA1* founder alleles [15]. Furthermore, *BRCA1* has a somewhat higher penetrance for HGSOC than *BRCA2* [18]. In accordance with other studies, the median age at diagnosis was evidently higher in *BRCA2* vs. *BRCA1* mutation carriers [19]. There were no statistically significant differences in the distribution of tumor stages between groups (Table 1). *BRCA2*-associated HGSOCs had a significantly higher rate of pCRs as compared to *BRCA1*-mutated cancers [8/15 (53%) vs. 7/48 (15%), Fisher’s exact test, two-tailed, *P* = 0.004]. When near-complete and complete responses were combined, the difference between *BRCA2*- and *BRCA1*-associated cancers remained significant [9/15 (60%) vs. 12/48 (25%), Fisher’s exact test, two-tailed, *P* = 0.02] (Table 1). The above calculations considered all patients included in the study. We further excluded 6 women, who did not undergo debulking surgery, either due to poor response to NACT or other reasons. This comparison produced an even more evident difference between *BRCA2* and *BRCA1* with regard to pCR [8/12 (75%) vs. 7/45 (16%); Fisher’s exact test, two-tailed, *P* = 0.0004]. Logistic regression analysis revealed that *BRCA2*-associated HGSOCs had a strikingly higher likelihood for CRS3 when compared to WT cases [OR = 14.0 (95% CI 4.0–48.4), *P* < 0.0001], while this difference was less pronounced for tumors arising in *BRCA1* mutation carriers [OR = 3.1 (95% CI 1.2–8.0), *P* = 0.02].

Our previous NACT investigations on patients with hereditary HGSOC demonstrated the superiority of the cisplatin and mitomycin C combination as compared to other schemes [20, 21]. Consequently, a substantial number of patients with *BRCA1*/*2*-associated HGSOC included in this study received this regimen, being a part of the NCT04747717 clinical trial. We further limited the comparison to patients who were treated with a “gold standard” doublet, i.e., carboplatin and paclitaxel (TCbP). This analysis produced an even more striking difference between *BRCA2* and *BRCA1* mutation carriers with regard to CRS3 [7/10 (70%) vs. 4/22 (18%), Fisher’s exact test, two-tailed, *P* = 0.007].

HGSOC patents of Slavic origin are highly enriched for the founder *BRCA1* c.5266dupC (5382insC) allele [22, 23]. Some studies indicate that the type of *BRCA* mutation may influence tumor responsiveness to chemotherapy [24]. Therefore, we compared NACT outcomes in patients carrying *BRCA1* c.5266dupC vs. women with other types of *BRCA1* pathogenic alleles. No significant differences were observed (Table 3).

**Table 3:** Localization of *BRCA1* mutation and CRS

**Mutation**	**No surgery**	**CRS1**	**CRS2**	**CRS3**	***P***
c.5266dupC (*n* = 24)	3 (13%)	3 (13%)	10 (42%)	8 (33%)	0.3
Other mutations (*n* = 24)	0 (0)	7 (26%)	13 (54%)	4 (17%)	

CRS: chemotherapy response score

### Receptor status rather than *BRCA1* or *BRCA2* mutation is a strong predictor of BC sensitivity to NACT

We further analyzed the frequency of pCR in BCs with distinct *BRCA* status. The analyzed groups were well-balanced with regard to tumor stages (Table 2). pCRs were observed in 38/69 (55%) *BRCA1* mutation carriers vs. 13/36 (36%) *BRCA2*-associated cancers (Fisher’s exact test, two-tailed, *P* = 0.1) or 43/150 (29%) *BRCA-*WT tumors (Fisher’s exact test, two-tailed, *P* = 0.0003). However, this comparison is of limited value because the above groups differed significantly from each other with regard to the proportion of patients with receptor triple-negative disease [*BRCA1*: 59/69 (86%); *BRCA2*: 10/36 (27%); WT: 76/150 (51%), Fisher’s exact test, *P* < 0.0001]. It is well established that triple-negative BCs are significantly more sensitive to NACT than tumors expressing steroid hormone receptors [11, 25]; therefore, we performed the comparison of the groups with different receptor statuses. The response rates in triple-negative patients were consistently higher than in HR+/HER2– patients across all analyzed subgroups [*BRCA1*: 35/59 (59%) vs. 3/10 (30%); *BRCA2*: 5/10 (50%) vs. 8/26 (31%); WT: 31/76 (41%) vs. 12/74 (16%); Mantel-Haenzsel test, *P* < 0.001].

**Figure 1:**
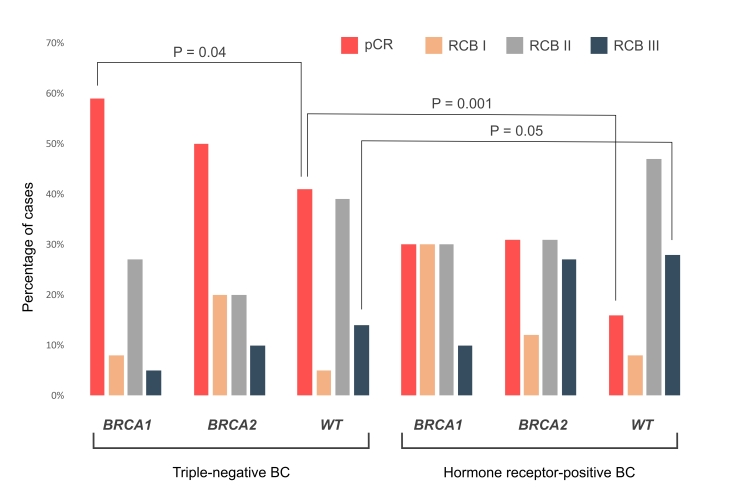
**RCB distribution in triple-negative and hormone receptor-positive carcinomas.** BC: breast cancer; pCR: pathologic complete response; RCB: residual cancer burden; WT: wild-type

It is of notice that the proportion of patients with unsatisfactory response to NACT (RCB III) was similarly high in both *BRCA2-*mutated [7/26 (27%)] and WT [21/74 (28%)] HR+ BCs (Figure 1 and Table S3). Overall, pCR was observed in 71/145 (49%) triple-negative vs. 23/110 (21%) HR+ BCs (Fisher’s exact test, two-tailed, *P* < 0.0001). The OR for achieving pCR was higher for receptor status (triple-negative vs. HR+: OR = 3.4, 95% CI 1.9–6.0, *P* < 0.001) than for BRCA status (any mutation vs. WT: OR = 2.1, 95% CI 1.2–3.6, *P* = 0.008).

The incorporation of carboplatin to the NACT is a common practice both in *BRCA1*/*2*-mutated patients and in women with triple-negative BC [11, 26–29]. In our dataset, 31/69 (45%) *BRCA1*-mutated, 11/36 (31%) *BRCA2*-mutated, and 26/150 (17%) WT patients received this drug. Strikingly, carboplatin did not improve pCR rates in *BRCA1*- or *BRCA2*-associated carcinomas. However, WT triple-negative BC patients receiving carboplatin showed a numerically higher frequency of pCR when compared to women treated by platinum-free regimens [14/26 (54%) vs. 15/44 (34%), Fisher’s exact test, two-tailed, *P* = 0.13].

## Discussion

*BRCA1*/*2* mutations account for approximately 25–30% of HGSOCs, with some studies providing even higher estimates [23, 30, 31]. *BRCA1*- and *BRCA2*-mutated HGSOCs are almost always discussed as a single entity, given that these categories of tumors do not have clear differences in clinical presentation, morphological and immunohistochemical appearance, or the pattern of chromosomal instability [32–34]. Only a few studies have compared treatment outcomes of *BRCA1* vs. *BRCA2* mutation carriers, and all these studies considered highly heterogeneous categories of HGSOC patients [8–10, 35]. This is the first study analyzing the immediate outcomes of NACT in *BRCA1*- vs. *BRCA2*-associated cancers. It demonstrates that *BRCA2*-associated HGSOCs have a higher rate of complete tumor cell elimination upon NACT when compared to *BRCA1*-mutated tumors. Although the size of this single-center study is small, the observed associations are in line with several other HGSOC investigations, which have demonstrated generally better survival for *BRCA2* vs. *BRCA1* mutation carriers [8, 9].

A number of biological mechanisms may underlie these differences. Although *BRCA1*/*2*-associated HGSOCs almost always carry a somatic deletion of the remaining allele of the gene (loss-of-heterozygosity, LOH) detected in the gross tumor mass, some tumors have a small admixture of platinum-resistant cells with a preserved WT copy of the involved *BRCA* gene. This explains the nature of post-NACT residual tumor masses: while short-term platinum therapy does not cause restoration of *BRCA1*/*2* open reading frame via a second mutation, the selection of pre-existing BRCA1/2-proficient tumor cells is observed at significant frequencies [36, 37]. Complete elimination of tumor cells upon NACT can be observed in HGSOCs, which do not have intratumoral heterogeneity with regard to LOH status. While intratumoral heterogeneity has already been demonstrated in *BRCA1*-related tumors, its involvement in the pathogenesis of *BRCA2*-associated cancers requires additional studies [38]. Some data suggest that *BRCA2* deficiency results in complete inactivation of DNA repair, while cells with biallelic *BRCA1* mutations still retain some DNA double-strand repair capacity due to the involvement of RAD51-driven pathways. Consequently, a higher level of sensitivity to platinum compounds and PARP inhibitors is generally observed in *BRCA2*- vs. *BRCA1*-defective cells [39]. Furthermore, taxanes are commonly included in NACT schemes for HGSOCs. Some laboratory and clinical studies suggest that taxanes are not effective against *BRCA1*-deficient cells, whereas *BRCA2* inactivation does not preclude the action of taxanes [2, 3, 40]. Our data strongly support these observations: indeed, the TCbP doublet produced only a moderate CRS3 rate in *BRCA1* mutation carriers, while excellent efficacy of this regimen was observed in *BRCA2*-associated HGSOCs.

Our study demonstrates that *BRCA1*-mutated BCs generally produce higher response rates to NACT as compared to *BRCA2*-related BCs. However, this difference is almost entirely attributed to the high frequency of triple-negative disease in *BRCA1* vs. *BRCA2* mutation carriers. When tumors were stratified according to receptor status, the NACT outcomes in the *BRCA1* and *BRCA2* groups appeared highly similar. Importantly, while *BRCA1*-mutated, *BRCA2-*mutated, and WT triple-negative tumors showed high rates of pCR, the frequency of NACT failure (i.e., RCB III) was similar between HR+ *BRCA2*-associated and WT cancers. It has to be acknowledged that pCR is a strong predictor of long-term survival only in triple-negative cancers, while the absence of pCR is not a potentially fatal indicator for HR+ disease [41, 42].

The high efficacy of single-agent platinum compounds has been convincingly demonstrated in a series of studies involving women with *BRCA1*/*2* germ-line mutations [43–45]. These data are in strong agreement with preclinical experiments [46, 47]. However, the rationale for the addition of carboplatin to conventional drug regimens is more controversial. Indeed, virtually all standard BC NACT regimens include anthracyclines. In general, platinum drugs and anthracyclines are both DNA double-strand-inducing agents and have a similar mode of action toward tumors with HRD [48, 49]. A randomized comparison of neoadjuvant cisplatin vs. doxorubicin-cyclophosphamide in *BRCA1*/*2* mutation carriers with BC did not reveal any differences between treatment arms [50]. Our study demonstrated that the addition of carboplatin did not improve the outcomes of NACT in *BRCA1*-mutated or *BRCA2*-mutated BCs, probably due to the redundancy of mechanisms of action of the involved drugs towards *BRCA*-deficient cells. This is in agreement with the results of the GeparSixto clinical trial, which involved triple-negative BC patients and subjected to separate analyses *BRCA1*/*2*-related and *BRCA1*/*2*-WT cases [26]. However, similar to the GeparSixto dataset, we observed a trend towards an improved pCR rate upon the addition of carboplatin in WT patients. This is an intriguing finding that deserves the analysis of the underlying biological mechanisms. *BRCA1*/*2*-WT BCs are a heterogeneous group of tumors, with some of them possibly having targets both for carboplatin and for conventional drug regimens.

Apparently, *BRCA1* and *BRCA2* mutations should not be regarded as equivalent factors when considering BC therapy. In addition to the role of HR status, other parameters, such as LOH for the remaining *BRCA* allele and the extent of HRD should be considered in future studies. Practical implementation of the upfront LOH and HRD testing may become complicated, as these techniques require a sufficient amount of tumor material and are not always compatible with BC biopsies.

NACT studies are highly informative because they involve therapy-naive patients, therefore, there are minimum factors potentially confounding treatment outcomes [12, 13]. Furthermore, they generally involve subjects with limited tumor spread, who are potentially amenable to surgery, i.e., variations in the tumor stages and patient comorbidities are usually small. This study suggests that ovarian carcinomas demonstrate better NACT outcomes in *BRCA2*- vs. *BRCA1*-mutated hereditary ovarian carcinomas, probably due to the higher sensitivity of the former to carboplatin-paclitaxel. The opposite trend is observed in BC, which is likely to be attributed to the high frequency of triple-negative disease in *BRCA1*- but not *BRCA2*-associated BCs.

The differences between the *BRCA1* and *BRCA2* mutations deserve further investigation. It is highly desirable to validate the above observations in large multi-center collections of *BRCA1*- and *BRCA2*-related tumors. At least two tendencies observed in the BC data set are potentially practice-changing and, therefore, require explicit clarification. First, HR status rather than *BRCA1*/*2* status has to be considered while discussing the feasibility of NACT. Second, the incorporation of carboplatin into the NACT scheme provides an advantage to *BRCA1*/*2* WT but not to *BRCA1*/*2*-mutated patients. Furthermore, the identification of biological mechanisms underlying clinical dissimilarities between *BRCA1*- and *BRCA2*-related tumors is of primary importance.

In conclusion, higher efficacy of NACT is observed in *BRCA2*- vs. *BRCA1*-associated OC. Steroid HR status but not *BRCA1*/*2* status is the strongest predictor of NACT efficacy in breast carcinomas.

## Abbreviations

BC: breast cancer
CRS: chemotherapy response score
HGSOCs: high-grade serous ovarian carcinomas
HR: hormone receptor
HRD: homologous recombination deficiency
LOH: loss-of-heterozygosity
NACT: neoadjuvant chemotherapy
OC: ovarian cancer
OR: odds ratio
pCR: pathologic complete response
RCB: residual cancer burden
TCbP: carboplatin and paclitaxel
WT: wild-type

## Supplementary materials

The supplementary Tables for this article are available at: https://www.explorationpub.com/uploads/Article/file/1002325_sup_1.xlsx.

## Declarations

### Availability of data and materials

All datasets for this study are included in the manuscript and the supplementary files.

### Copyright

© The Author(s) 2025.
